# Associations of non-high-density lipoprotein cholesterol to high-density lipoprotein cholesterol ratio with diabetes and prediabetes among adults with hypertension: a cross-sectional study

**DOI:** 10.3389/fendo.2025.1523016

**Published:** 2025-05-08

**Authors:** Qing Meng, Shengqiang Fan, Li Zhang, Bin Shen, Chaoping Zou, Dezhou Sun, Xianghui Liu, Jian Zhang, Shugang Xu

**Affiliations:** ^1^ School of Clinical Medicine, Shandong Second Medical University, Weifang, China; ^2^ Neurosurgery Department, Dezhou People's Hospital, Dezhou, Shandong, China; ^3^ Clinical College of Neurology, Neurosurgery and Neurorehabilitation, Tianjin Medical University, Tianjin, China

**Keywords:** NHANES, NHHR, diabetes, prediabetes, hypertension

## Abstract

**Background:**

The non-high-density lipoprotein cholesterol to high-density lipoprotein cholesterol ratio (NHHR) is an emerging composite lipid marker. Prediabetes, characterized by an asymptomatic state with moderate hyperglycemia, is more prevalent than diabetes. This study aimed to elucidate the potential correlation between NHHR and the risk of diabetes and prediabetes among adults with hypertension.

**Methods:**

In this cross-sectional survey, we screened National Health and Nutrition Examination Survey (NHANES)-collected data during 2009-2018, identifying a qualifying population of 10,250 individuals. Weighted multivariate logistic regression and curve fitting evaluated the correlation between the NHHR and the incidence of diabetes and prediabetes. To test differences between subgroups, stratified analyses were performed. Additionally, prediction accuracy of the NHHR was assessed using receiver operating characteristic (ROC) curves.

**Results:**

We included 10,250 patients with hypertension (mean age, 56.31 ± 16.06 years) including 2,198 with diabetes and 4,138 with prediabetes—a combined prevalence of 61.81%. The fully adjusted model indicated each unit increase in NHHR was associated with a 21% higher risk of diabetes/prediabetes (OR 1.21; 95% CI, 1.15-1.25). Adjustment using multivariable classification models revealed that compared to the lowest NHHR quartile, the odds increased by 41% (OR 1.37; 95% CI, 1.27-1.59, p<0.001) in Q3 and (OR 1.82; 95% CI, 1.62-1.98, p<0.001) in Q4. In patients with hypertension, the NHHR was positively correlated with the prevalence of diabetes and prediabetes, with a nonlinear trend in the fitted curve (nonlinearity, P=0.007). The threshold effect analysis showed that the inflection point for NHHR and the risk of diabetes and prediabetes was 7.09. In particular, when NHHR was below 7.09, a positive correlation was found between NHHR and the risk of diabetes and prediabetes in this population (OR 1.34; 95% CI, 1.28–1.39). Subgroup analyses showed consistent associations across most groups, with a significant interaction in sex.

**Conclusions:**

NHHR is positively and non-linearly correlated with diabetes/prediabetes in patients with hypertension, particularly among women. It may serve as a valuable tool for early risk assessment and management.

## 1 Introduction

Diabetes, as one of the most common metabolic diseases, has become a major burden on global healthcare system (1), with approximately 6.7 million deaths attributed to it. According to data released by the International Diabetes Federation, the prevalence of diabetes is expected to rise to 590 million by 2030 (2). Global statistics estimate that diabetes currently causes around 2.4 million deaths annually. Moreover, people with diabetes typically have a life expectancy that is 6-8 years shorter than those without the condition, making diabetes the seventh leading cause of death worldwide (3). Prediabetes is a condition in which blood glucose levels are elevated above the standard range but remain below the diagnostic threshold for diabetes. It is widely considered a precursor to type 2 diabetes, which may progress to full-fledged diabetes within a few years if not properly managed. Each year, about 5-10% of individuals with prediabetes develop prediabetes, and by 2030, it is projected that over 470 million people could be affected (4). Prediabetes can impair kidney filtration function owing to prolonged hyperglycemia, potentially leading to kidney failure. It also increases the risk of cardiovascular diseases such as coronary heart disease, stroke, and hypertension (5, 6). The underlying mechanisms are mainly related to insulin resistance and pancreatic beta cell dysfunction (7). Therefore, it is crucial to prioritize strategies for the prevention and treatment of both diabetes and prediabetes.

NHHR is considered a composite lipid marker commonly used to reflect lipid metabolism status and assess cardiovascular health (8). It has shown excellent performance in diagnosing and predicting hyperlipidemia and atherosclerosis (9, 10). Within NHHR, low-density lipoprotein cholesterol (LDL-C) typically accounts for 50-70% of non-HDL-C, while high-density lipoprotein cholesterol (HDL-C), a protective factor, is responsible for the reverse transport and clearance of excess cholesterol from tissues. Elevated NHHR levels have been linked to increased risk for a variety of diseases, including depression, kidney stones, and breast cancer (11–13).

Compared to other lipid markers, NHHR offers superior predictive value for cardiovascular disease, metabolic syndrome, fatty liver, and certain renal diseases. Diabetes is frequently associated with abnormal lipid profiles (14, 15). Studies have shown that decreased HDL-C levels and elevated LDL-C and triglycerides (TG) levels are independently and significantly related to the development of diabetes (16–18). A retrospective study involving 41,821 participants from the Korean population found that the non-HDL-C/HDL-C ratio is more strongly associated with insulin resistance (IR) than the apo B/apo A1 ratio. Therefore, we hypothesized that NHHR is positively correlated with the risk of prediabetes. However, research specifically exploring the correlation between NHHR and diabetes incidence remains limited, particularly regarding prediabetes and specific demographic groups. Therefore, the aim of this study was to investigate the association between NHHR and the risk of diabetes and prediabetes in individuals with hypertension. This may support the use of NHHR as a valuable tool for identifying individuals at high risk for these conditions.

## 2 Materials and methods

### 2.1 Study population

**Figure 1:**
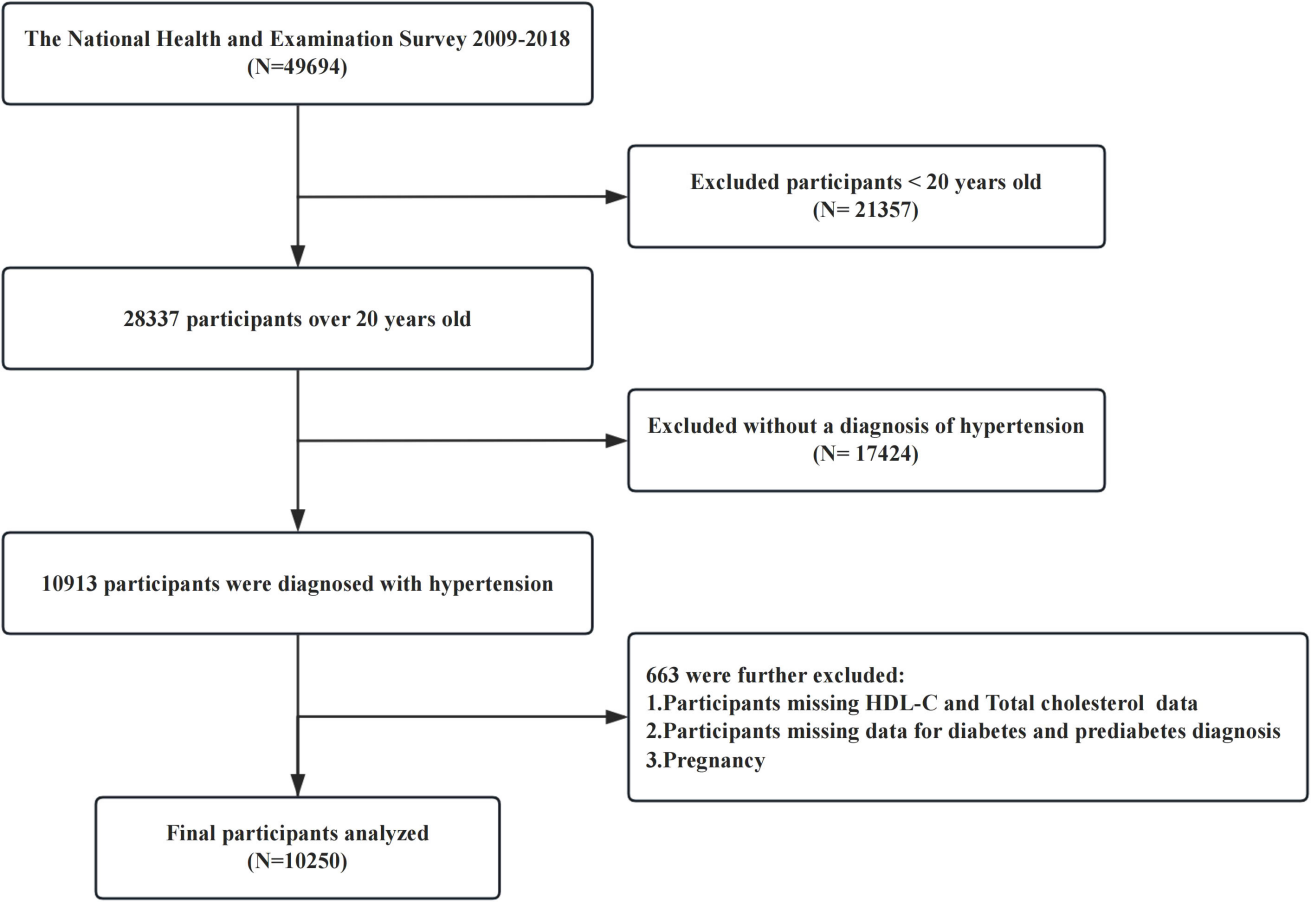
Flowchart of participants included.

The NHANES uses a multistage, stratified, and clustered sampling design to collect nationally representative health examination data from the US population, including basic information on participants, laboratory test results, and underlying diseases. The NHANES protocol was approved by the Ethics Review Committee of the National Center for Health Statistics (NCHS), and written consent was obtained from each participant prior to their involvement in the study. The research procedures were authorized by the NCHS Ethics Review Board, and the study complied with the ethical standards set forth in the Declaration of Helsinki. For this cross-sectional study, we used data from five cycles of the NHANES (2009-2018), that included a cohort of 49,694 participants. Our study excluded individuals under 20 years of age (n = 21,357), participants without a diagnosis of hypertension (n = 17,424), those with missing data on high-density lipoprotein cholesterol or total cholesterol (TC) levels, those lacking diagnostic criteria for diabetes or prediabetes, and pregnant or lactating females. After these exclusions, 10,250 participants were included in the final analysis (**Figure 1**).

Sample collection followed strict standardized procedures conducted at Mobile Examination Centers. Following venous blood collection, samples were transported to the central laboratory for analysis. If testing could not be conducted within the designated time frame, samples were either refrigerated or frozen at appropriate temperatures. All experimental procedures were performed by trained professionals in accordance with standardized protocols (19).

### 2.2 Diagnosis of hypertension

Hypertension was assessed by averaging three–two blood pressure measurements. If only one measurement was available, the use of antihypertensive medication was also considered. Individuals were classified as hypertensive if their systolic blood pressure was ≥130 mmHg and/or diastolic blood pressure was ≥80 mmHg. Blood pressure measurements are conducted by trained technicians using either mercury sphygmomanometers or electronic blood pressure devices, following established international guidelines (20).

### 2.3 Measurement of NHHR

The exposure variable, NHHR, was calculated as non-HDL-C divided by HDL-C (21). Non-HDL-C was determined by subtracting HDL-C from TC. HDL-C and TC concentrations were measured using precipitation or immunoassay techniques. Participants were divided into four groups (Q1, Q2, Q3, and Q4) based on NHHR quartiles, with Q1 serving as the reference group.

### 2.4 Diagnosis of diabetes and prediabetes

According to international guidelines and prior studies (22), diabetes was diagnosed based on: 1) physician-conformed diagnosis; 2) fasting blood glucose (FPG) levels ≥7.0 mmol/L; 3) hemoglobin A1c (HbA1c) ≥6.5%; 4) use of anti-diabetic medication or insulin. Prediabetes was defined as (3): self-reported prediabetes, 5.7% ≤ HbA1c <6.5%, or FPG levels ranged from 5.6 to 7.0 mmol/L.

### 2.5 Covariable screening

Covariate selection was based on existing literature and clinical expertise, considering multiple confounders that may affect NHHR and diabetes risk. Demographic variables included age, sex, ethnicity, educational level, marital status, family income-to-poverty ratio (PIR), and body mass index (BMI; 18.5–25 kg/m², 25–30 kg/m², ≥30 kg/m²). Lifestyle variables included smoking status (never, former, current), alcohol consumption (yes/no), and physical activity (PA). Alcohol use was defined as consumption of at least 12 alcoholic beverages annually. PA was categorized using total metabolic equivalent (MET) minutes per week, following U.S. Physical Activity Guidelines: inactive (0 MET-min/week), insufficiently active (<600 MET-min/week), and active (>600 MET-min/week). Laboratory measures included albumin, HbA1c, total cholesterol (TC), triglyceride (TG), HDL-C, serum creatinine (SCR), and uric acid. Comorbidities included cardiovascular disease (CVD) and chronic kidney disease (CKD), with CKD defined as self-reported CKD, eGFR <60 ml/min/1.73 m², or urine albumin-to-creatinine ratio (UACR) ≥30 mg/g.

### 2.6 Statistical analysis

Statistical analyses were performed using R software (version 4.1.0) and Free Statistics software. NHANES weights were applied according to guidelines, with data spanning five cycles (2009-2018) and adjusted using WTMEC2YR/5. Continuous variables are presented as means ± standard deviations (SD), while categorical variables are expressed as percentages. Group differences by NHHR quartile were assessed using chi-square tests for categorical variables, Kruskal–Wallis test for non-normally distributed continuous variables, and t-tests for normally distributed variables. Weighted logistic regression models were used to estimate odds ratios (OR) and 95% confidence intervals (CI) for the association between NHHR and diabetes/prediabetes risk. Model 1 was unadjusted; Model 2 adjusted for demographic variables (sex, age, education, race, marital status, and PIR); and Model 3 included additional adjustments for BMI, waist circumference, smoking, alcohol use, PA, uric acid, CKD, and CVD. Restricted cubic spline (RCS) curves and threshold effect analyses, based on Model 3 covariates, were used to evaluate potential nonlinear associations between NHHR and diabetes/prediabetes in patients with hypertension. Stratified analysis were conducted by age, sex, race, BMI, CKD, CVD, smoking status, alcohol use, and PA. ROC curves evaluated NHHR’s predictive value in identifying diabetes and prediabetes in this population. To enhance statistical efficiency and minimize bias, multiple imputations was applied to handle missing data. Sensitivity analysis were conducted to validate the stability of the results, revealing no significant differences between the imputed and original datasets. A two-tailed P-value less than 0.05 was considered statistically significant.

## 3 Results

### 3.1 Baseline characteristics of participants

Our study included 10,250 patients with hypertension, comprising 5,561 males and 4,689 females, with a mean age of 56.31 ± 16.06 years. The overall prevalence of diabetes and prediabetes was 61.81%, with 2,198 and 4,138 individuals having diabetes and prediabetes, respectively. The participants were categorized into four groups according to NHHR quartiles: Q1 ≤ 1.98, 1.98 < Q2 ≤ 2.79, 2.79 < Q3 ≤ 3.79, and 3.79 < Q4. **Tables 1** and **2** describe the baseline characteristics of the participants based on NHHR quartile and disease status, respectively. The mean (SD) of NHHR was markedly elevated in individuals with diabetes and prediabetes, at 3.14 (1.51), compared to a mean (SD) of 2.88 (1.43) in those without prediabetes. Participants with a history of diabetes or prediabetes were generally older. In terms of lifestyle, the diabetes and prediabetes groups had higher BMI and waist circumference, as well as less frequent PA. Additionally, this group exhibited more than double the prevalence of CKD and CHD compared with that of the non-prediabetes group. As the NHHR quartiles increased, the proportion of individuals with diabetes and prediabetes increased significantly (from 55.78% to 67.43%). However, there was no significant sex differences in disease prevalence between males and females.

**Table 1:** Baseline characteristics of the study population divided by different disease states.

Variables	Total	None prediabetes	Diabetes and prediabetes	*P*-value
Participants	10250	3914	6336	
Sex, n (%)				0.337
Male	5561 (54.25)	2147 (54.85)	3414 (53.88)	
Female	4689 (45.75)	1767 (45.15)	2922 (46.12)	
Age, Mean ± SD	56.31 ± 16.06	50.44 ± 16.79	59.94 ± 14.45	< 0.001
Race, n (%)				< 0.001
Mexican American	1343 (13.10)	451 (11.52)	892 (14.08)	
Other Hispanic	1016 ( 9.91)	358 (9.15)	658 (10.39)	
Non-Hispanic White	3995 (38.98)	1749 (44.69)	2246 (35.45)	
Non-Hispanic Black	2566 (25.03)	851 (21.74)	1715 (27.07)	
Other Race	1330 (12.98)	505 (12.9)	825 (13.02)	
Education, n (%)				< 0.001
Under high school	2663 (25.98)	794 (20.29)	1869 (29.5)	
High school or equivalent	2416 (23.57)	931 (23.79)	1485 (23.44)	
Above high school	5161 (50.35)	2187 (55.88)	2974 (46.94)	
Marriage, n (%)				< 0.001
Never married	1399 (13.65)	710 (18.14)	689 (10.87)	
Married or living with a partner	6068 (59.20)	2333 (59.61)	3735 (58.95)	
Other	2783 (27.15)	871 (22.25)	1912 (30.18)	
PIR, n (%)				< 0.001
<1	1932 (20.87)	704 (19.69)	1228 (21.62)	
1-3	4070 (43.97)	1501 (41.99)	2569 (45.22)	
>3	3254 (35.16)	1370 (38.32)	1884 (33.16)	
BMI(kg/m2), n (%)				< 0.001
Normal (18.5–25)	2322 (22.89)	1176 (30.31)	1146 (18.3)	
Overweight (25–30)	3346 (32.99)	1324 (34.12)	2022 (32.28)	
Obesity (≥30)	4476 (44.12)	1380 (35.57)	3096 (49.43)	
Waist circumference (cm), Mean ± SD	102.84 ± 16.18	98.67 ± 15.41	105.46 ± 16.11	< 0.001
Smoking status, n (%)				< 0.001
Never smoker	5501 (53.67)	2177 (55.62)	3324 (52.46)	
Former smoker	2810 (27.41)	912 (23.3)	1898 (29.96)	
Current smoker	1939 (18.92)	825 (21.08)	1114 (17.58)	
Drinking status, n (%)				< 0.001
Nondrinker	2861 (29.88)	935 (25.58)	1926 (32.53)	
drinker	6715 (70.12)	2720 (74.42)	3995 (67.47)	
Physical Activity, n (%)				< 0.001
inactive	6224 (60.72)	2258 (57.69)	3966 (62.59)	
insufficiently active	775 ( 7.56)	285 (7.28)	490 (7.73)	
Sufficiently active	3251 (31.72)	1371 (35.03)	1880 (29.67)	
Total cholesterol (mmol/L), Mean ± SD	5.09 ± 1.10	5.12 ± 1.04	5.06 ± 1.14	0.006
Triglycerides (mmol/L), Mean ± SD	1.88 ± 1.47	1.69 ± 1.28	2.00 ± 1.57	< 0.001
HDL-C (mmol/L), Mean ± SD	1.88 ± 1.47	1.69 ± 1.28	2.00 ± 1.57	< 0.001
NHHR, Mean ± SD	3.04 ± 1.49	2.88 ± 1.43	3.14 ± 1.51	< 0.001
Uric acid (umol/L), Mean ± SD	338.35 ± 85.76	327.93 ± 82.86	344.79 ± 86.88	< 0.001
eGFR, Mean ± SD	88.15 ± 22.39	93.85 ± 20.84	84.63 ± 22.59	< 0.001
UACR, Mean ± SD	77.10 ± 501.30	41.95 ± 461.97	98.91 ± 523.08	< 0.001
CKD, n (%)	2536 (24.74)	568 (14.51)	1968 (31.06)	< 0.001
CVD, n (%)	1113 (13.67)	241 (7.67)	872 (17.44)	< 0.001

Mean ± SD, for continuous variables: the p-value was analyzed via ANOVA. (%) for categorical variables: the p-value was analyzed via the weighted chi-square test.

NHHR, the ratio of non-high-density lipoprotein cholesterol to high-density lipoprotein cholesterol; PIR, The ratio of family income to poverty; BMI, Body Mass Index; eGFR: estimated glomerular filtration rate; UACR:urine albumin-to-creatinine ratio; CKD,chronic kidney disease; CVD cardiovascular disease.

**Table 2:** Baseline characteristics of the study population divided by NHHR Quartile.

Variables	Total	Q1 (NHHR≤ 1.98)	Q2 (1.98<NHHR≤2.79)	Q3 (2.79<NHHR≤3.79)	Q4 (NHHR>3.79)	*P*-value
Participants	10250	2551	2571	2564	2564	
Sex, n (%)						< 0.001
Male	5561 (54.25)	1102 (43.2)	1219 (47.41)	1469 (57.29)	1771 (69.07)	
Female	4689 (45.75)	1449 (56.8)	1352 (52.59)	1095 (42.71)	793 (30.93)	
Age, Mean ± SD	56.31 ± 16.06	59.76 ± 16.71	58.48 ± 15.92	54.87 ± 15.55	52.14 ± 14.88	< 0.001
Race, n (%)						< 0.001
Mexican American	1343 (13.10)	241 (9.45)	315 (12.25)	377 (14.7)	410 (15.99)	
Other Hispanic	1016 ( 9.91)	168 (6.59)	249 (9.68)	290 (11.31)	309 (12.05)	
Non-Hispanic White	3995 (38.98)	999 (39.16)	989 (38.47)	975 (38.03)	1032 (40.25)	
Non-Hispanic Black	2566 (25.03)	813 (31.87)	719 (27.97)	575 (22.43)	459 (17.9)	
Other Race	1330 (12.98)	330 (12.94)	299 (11.63)	347 (13.53)	354 (13.81)	
Education, n (%)						0.004
Under high school	2663 (25.98)	637 (24.97)	654 (25.44)	657 (25.62)	715 (27.89)	
High school or equivalent	2416 (23.57)	569 (22.3)	591 (22.99)	648 (25.27)	608 (23.71)	
Above high school	5161 (50.35)	1340 (52.53)	1325 (51.54)	1259 (49.1)	1237 (48.24)	
Marriage, n (%)						< 0.001
Never married	1399 (13.65)	383 (15.01)	337 (13.11)	346 (13.49)	333 (12.99)	
Married or living with a partner	6068 (59.20)	1335 (52.33)	1489 (57.92)	1581 (61.66)	1663 (64.86)	
Other	2783 (27.15)	833 (32.65)	745 (28.98)	637 (24.84)	568 (22.15)	
PIR, n (%)						0.038
<1	1932 (20.87)	476 (20.7)	470 (20.3)	471 (20.37)	515 (22.11)	
1-3	4070 (43.97)	979 (42.57)	992 (42.85)	1051 (45.46)	1048 (45)	
>3	3254 (35.16)	845 (36.74)	853 (36.85)	790 (34.17)	766 (32.89)	
BMI(kg/m2), n (%)						< 0.001
Normal (18.5–25)	2322 (22.89)	1022 (40.51)	599 (23.55)	412 (16.21)	289 (11.4)	
Overweight (25–30)	3346 (32.99)	778 (30.84)	844 (33.19)	868 (34.15)	856 (33.75)	
Obesity (≥30)	4476 (44.12)	723 (28.66)	1100 (43.26)	1262 (49.65)	1391 (54.85)	
Waist circumference (cm), Mean ± SD	102.84 ± 16.18	95.97 ± 15.98	102.60 ± 15.97	105.36 ± 15.68	107.31 ± 14.78	< 0.001
Diabetes, n (%)	2198 (21.44)	475 (18.62)	534 (20.77)	561 (21.88)	628 (24.49)	< 0.001
Prediabetes, n (%)	4138 (40.37)	948 (37.16)	1034 (40.22)	1055 (41.15)	1101 (42.94)	< 0.001
Diabetes and prediabetes, n (%)	6336 (61.81)	1423 (55.78)	1568 (60.99)	1616 (63.03)	1729 (67.43)	< 0.001
Smoking status, n (%)						< 0.001
Never smoker	5501 (53.67)	1394 (54.65)	1432 (55.7)	1402 (54.68)	1273 (49.65)	
Former smoker	2810 (27.41)	684 (26.81)	737 (28.67)	707 (27.57)	682 (26.6)	
Current smoker	1939 (18.92)	473 (18.54)	402 (15.64)	455 (17.75)	609 (23.75)	
Drinking status, n (%)						< 0.001
Nondrinker	2861 (29.88)	686 (29.04)	791 (33.07)	743 (30.84)	641 (26.56)	
drinker	6715 (70.12)	1676 (70.96)	1601 (66.93)	1666 (69.16)	1772 (73.44)	
Physical Activity, n (%)						< 0.001
inactive	6224 (60.72)	1620 (63.5)	1635 (63.59)	1500 (58.5)	1469 (57.29)	
insufficiently active	775 ( 7.56)	197 (7.72)	183 (7.12)	191 (7.45)	204 (7.96)	
sufficiently active	3251 (31.72)	734 (28.77)	753 (29.29)	873 (34.05)	891 (34.75)	
Total cholesterol (mmol/L), Mean ± SD	5.09 ± 1.10	4.50 ± 0.96	4.81 ± 0.93	5.17 ± 0.90	5.86 ± 1.11	< 0.001
Triglycerides (mmol/L), Mean ± SD	1.88 ± 1.47	1.05 ± 0.51	1.43 ± 0.65	1.88 ± 0.91	3.14 ± 2.16	< 0.001
HDL-C (mmol/L), Mean ± SD	1.37 ± 0.44	1.82 ± 0.47	1.43 ± 0.29	1.22 ± 0.22	1.00 ± 0.20	< 0.001
Uric acid (umol/L), Mean ± SD	338.35 ± 85.76	312.24 ± 82.17	330.11 ± 82.27	345.54 ± 83.07	365.31 ± 86.46	< 0.001
eGFR, Mean ± SD	88.15 ± 22.39	85.35 ± 23.09	85.95 ± 22.36	89.54 ± 22.00	91.75 ± 21.49	< 0.001
UACR, Mean ± SD	77.10 ± 501.30	65.89 ± 410.10	62.96 ± 352.45	80.69 ± 605.00	98.70 ± 587.38	0.044
CKD, n (%)	2536 (24.74)	679 (26.62)	663 (25.79)	611 (23.83)	583 (22.74)	0.005
CVD, n (%)	1113 (13.67)	342 (17.28)	278 (13.63)	251 (12.4)	242 (11.52)	< 0.001

Mean ± SD, for continuous variables: the p-value was analyzed via ANOVA. (%) for categorical variables: the p-value was analyzed via the weighted chi-square test.

NHHR, the ratio of non-high-density lipoprotein cholesterol to high-density lipoprotein cholesterol; PIR, The ratio of family income to poverty; BMI, Body Mass Index; eGFR, estimated glomerular filtration rate; UACR, urine albumin-to-creatinine ratio; CKD, chronic kidney disease; CVD, cardiovascular disease.

### 3.2 Univariate analysis

Univariate logistic regression analysis revealed increased risk of diabetes and prediabetes with higher NHHR and non-HDL-C levels (NHHR: OR 1.13; 95% CI, 1.10-1.17; non-HDL-C: OR 1.05; 95% CI, 1.02-1.09). Individuals with a PIR > 3 had a lower risk of both the conditions than those with a PIR < 1. In addition, age, BMI, waist circumference, uric acid, triglycerides, CKD, and CVD were positively associated with the occurrence of diabetes and prediabetes, whereas HDL-C level, sufficient PA, and PIR were negatively correlated with both the conditions in this population (**Table 3**).

**Table 3:** Univariate logistic regression analysis of association between NHHR and the risk of diabetes and prediabetes in adults with hypertension.

Variables	OR (95%CI)	P-value
Sex		
Male	1(Ref)	
Female	1.04 (0.96~1.13)	0.337
Age	1.04 (1.04~1.04)	< 0.001
Race		< 0.001
Mexican American	1(Ref)	
Other Hispanic	0.93 (0.78~1.1)	0.402
Non-Hispanic White	0.65 (0.57~0.74)	<0.001
Non-Hispanic Black	1.02 (0.89~1.17)	0.793
Other Race	0.83 (0.7~0.97)	0.018
Education		< 0.001
< high school	1(Ref)	
High school	0.68 (0.6~0.76)	< 0.001
> high school	0.58 (0.52~0.64)	<0.001
Marriage		
Never married	1(Ref)	
Married	1.65 (1.47~1.85)	< 0.001
Other	2.26 (1.98~2.58)	< 0.001
PIR	0.93 (0.91~0.96)	< 0.001
<1	1(Ref)	
1-3	0.98 (0.88~1.1)	0.741
>3	0.79 (0.7~0.89)	<0.001
BMI	1.05 (1.04~1.06)	< 0.001
Waist circumference	1.13 (1.10~1.17)	< 0.001
NHHR	2.51 (1.89~3.32)	< 0.001
non-HDL-C	1.05 (1.02~1.09)	0.012
HDL	0.53 (0.49~0.59)	< 0.001
Smoking status		
Never smoker	1(Ref)	
Former smoker	1.36 (1.24~1.5)	< 0.001
Current smoker	0.88 (0.8~0.98)	0.022
Drinking status		
Nondrinker	1(Ref)	
drinker	0.71 (0.65~0.78)	< 0.001
Physical Activity		
inactive	1(Ref)	
insufficiently active	0.98 (0.84~1.14)	0.787
sufficiently active	0.78 (0.72~0.85)	< 0.001
CKD		
no	1(Ref)	
yes	2.65 (2.39~2.94)	< 0.001
CVD		
no	1(Ref)	
yes	2.54 (2.19~2.96)	< 0.001
Uric acid	1.23 (1.18~1.29)	< 0.001

OR, odds ratio; 95% CI, 95% confidence interval.

NHHR, the ratio of non-high-density lipoprotein cholesterol to high-density lipoprotein cholesterol; PIR, The ratio of family income to poverty; BMI, Body Mass Index; eGFR, estimated glomerular filtration rate; UACR, urine albumin-to-creatinine ratio; CKD, chronic kidney disease; CVD, cardiovascular disease.

### 3.3 Associations between NHHR and risk of diabetes/prediabetes

As shown in **Table 4**, we assessed the correlation between NHHR and diabetes/prediabetes in the hypertensive population using three logistic regression models, with the effect size represented by the OR and 95%CI. In the crude model, every unit increase in NHHR correlated with a 13% increase in the risk of diabetes/prediabetes (OR 1.13, 95% CI 1.10–1.17, P < 0.001). After analyzing NHHR as a continuous variable and adjusting for multiple potential confounders (Model 3), the multivariate logistic regression model produced results that were not significantly different from those of the unadjusted model (OR 1.21; 95% CI, 1.15-1.25, P < 0.001). Furthermore, when we converted NHHR into categorical variables based on quartiles, univariate analyses showed that individuals in the highest quartile had a significantly higher likelihood of diabetes and prediabetes compared to those in the lowest quartile (Q4 OR, 1.64; 95%CI, 1.46-1.84, P < 0.001). Adjustments performed using multivariate models revealed a 37% and 82% increase in the prevalence of diabetes and prediabetes in Q3 (OR 1.37; 95% CI, 1.27-1.59, P < 0.001) and Q4 (OR 1.82; 95% CI, 1.62-1.98, P < 0.001), using Q1 as the reference group. Thus, NHHR may serve as a potential predictor of diabetes and prediabetes in this population. **Tables 5** presents the association between NHHR and the risk of diabetes and prediabetes in both male and female patients. The data revealed that as NHHR increases, the risk of developing diabetes/prediabetes is significantly higher in women compared to men (Female: OR 1.32; 95% CI, 1.23-1.41; Male: OR 1.16; 95% CI, 1.11-1.23).

**Table 4:** Multivariate Logistic analysis of association between NHHR and the risk of diabetes/prediabetes in adults with hypertension.

Variable	Model 0		Model 1		Model 2	
	OR (95%CI)	*P*-value	OR (95%CI)	*P*-value	OR (95%CI)	*P*-value
NHHR continues	1.13 (1.10~1.17)	<0.001	1.29 (1.25~1.34)	<0.001	1.21 (1.15~1.25)	<0.001
NHHR quartiles						
Q1	Reference		Reference		Reference	
Q2	1.24 (1.11~1.39)	<0.001	1.37 (1.21~1.54)	<0.001	1.14 (0.97~1.34)	0.102
Q3	1.35 (1.21~1.51)	<0.001	1.61 (1.43~1.85)	<0.001	1.37 (1.27~1.59)	<0.001
Q4	1.64 (1.46~1.84)	<0.001	2.35 (2.15~2.56)	<0.001	1.82 (1.62~1.98)	<0.001
*p* for trend	<0.001		<0.001		<0.001	

OR, odds ratio; 95% CI, 95% confidence interval; NHHR, the ratio of non-high-density lipoprotein cholesterol to high-density lipoprotein cholesterol.

Model 1: Non-adjusted.

Model 2: Sex, age, education, race, marital status, and PIR.

Model 3: Model 2 + BMI, Waist circumference, smoking status, drinking habits, physical activity, Uric acid, CVD, CKD.

**Table 5A:** Multivariate Logistic analysis of association between NHHR and the risk of diabetes/prediabetes in the male hypertension population.

Variable	Model 0		Model 1		Model 2	
	OR (95%CI)	*P*-value	OR (95%CI)	*P*-value	OR (95%CI)	*P*-value
NHHR continues	1.09 (1.05~1.13)	<0.001	1.23 (1.18~1.29)	<0.001	1.16 (1.11~1.23)	<0.001
NHHR quartiles						
Q1	Reference		Reference		Reference	
Q2	1.22 (1.05~1.42)	0.009	1.37 (1.15~1.63)	<0.001	1.26 (1.01~1.55)	0.037
Q3	1.22 (1.05~1.43)	0.009	1.75 (1.47~2.09)	<0.001	1.33 (1.06~1.65)	0.012
Q4	1.45 (1.24~1.69)	<0.001	2.32 (2.01~2.68)	<0.001	1.89 (1.61~2.26)	<0.001
*p* for trend	<0.001		<0.001		<0.001	

OR, odds ratio; 95% CI, 95% confidence interval.

Model 1: Non-adjusted.

Model 2: Age, education, race, marital status, and PIR.

Model 3: Model 2 + BMI, Waist circumference, smoking status, drinking habits, physical activity, Uric acid, CVD, CKD.

**Table 5B:** Multivariate Logistic analysis of association between NHHR and the risk of diabetes/prediabetes in the female hypertension population.

Variable	Model 0		Model 1		Model 2	
	OR (95%CI)	*P*-value	OR (95%CI)	*P*-value	OR (95%CI)	*P*-value
NHHR continues	1.26 (1.2~1.33)	<0.001	1.39 (1.31~1.48)	<0.001	1.32 (1.23~1.41)	<0.001
NHHR quartiles						
Q1	Reference		Reference		Reference	
Q2	1.22 (1.02~1.42)	0.027	1.37 (1.14~1.64)	0.001	1.34 (1.07~1.67)	0.012
Q3	1.35 (1.14~1.59)	<0.001	1.55 (1.28~1.87)	<0.001	1.46 (1.18~1.72)	0.008
Q4	1.96 (1.66~2.33)	<0.001	2.56 (2.07~2.86)	<0.001	2.15 (1.66~2.48)	<0.001
*p* for trend	<0.001		<0.001		<0.001	

OR, odds ratio; 95% CI, 95% confidence interval.

Model 1: Non-adjusted.

Model 2: Age, education, race, marital status, and PIR.

Model 3: Model 2 + BMI, Waist circumference, smoking status, drinking habits, physical activity, Uric acid, CVD, CKD.

### 3.4 Nonlinear relationships between NHHR and prediabetes/diabetes

**Figure 2:**
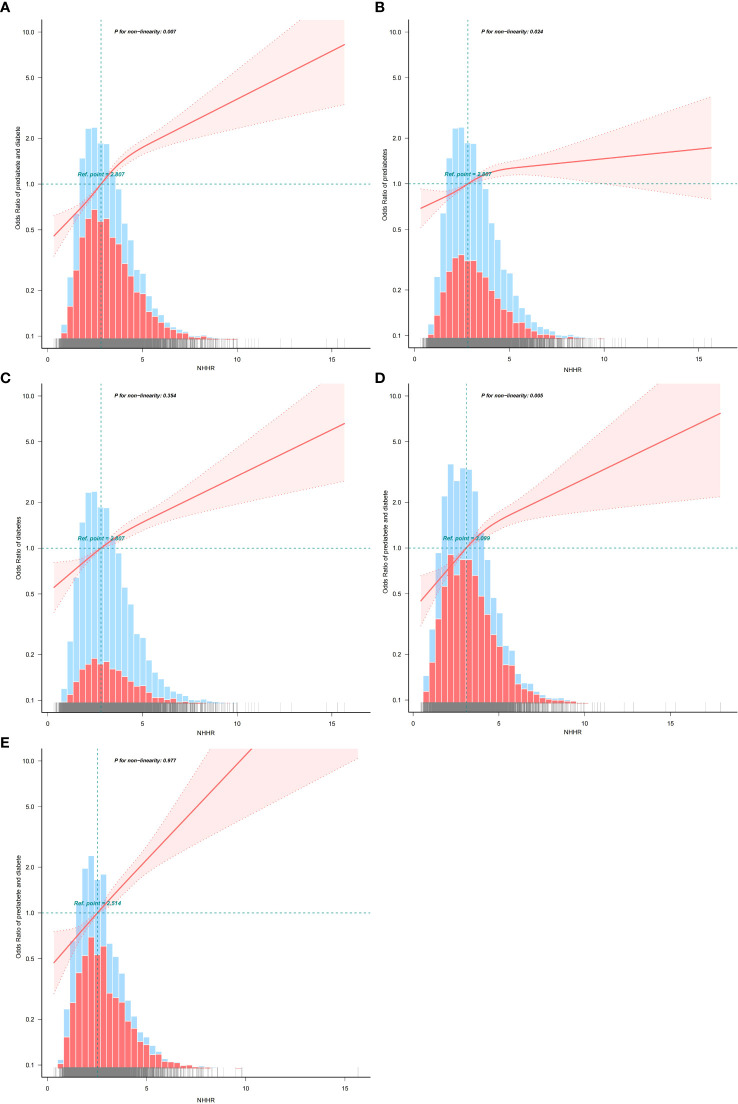
**(A)** The association between NHHR and the risk of diabetes/prediabetes in hypertension population. **(B)** The association between NHHR and the risk of prediabetes in hypertension population. **(C)** The association between NHHR and the risk of diabetes in hypertension population. **(D)** The association between NHHR and the risk of diabetes/prediabetes in the male hypertension population. **(E)** The association between NHHR and the risk of diabetes/prediabetes in the female hypertension population.

Using RCS analysis, we further explored the correlation between NHHR and diabetes/prediabetes in a hypertensive population stratified by sex. After adjusting for relevant covariates, the RCS analysis revealed a nonlinear association between the NHHR and diabetes/prediabetes (nonlinear P = 0.007). Threshold effect analysis identified a cutoff point at NHHR = 7.09. Below this threshold, the OR was 1.34 (95% CI: 1.28–1.39, P < 0.001), indicating a strong positive association (**Table 6**). In the sex-stratified analysis, a significant nonlinear relationship was observed in males (nonlinear P = 0.005), while the association appeared linear among females (nonlinear P = 0.997) (**Figure 2**).

**Table 6:** Threshold effect analysis of NHHR on diabetes and prediabetes.

NHHR	OR (95%CI)	*P*-value
E BK1	7.09 (6.89 ,7.28)	
slope1	1.34 (1.28~1.39)	<0.001
slope2	1.01 (0.81~1.26)	0.4849
Likelihood Ratio test		0.007

### 3.5 Stratified analyses and sensitivity analysis

**Figure 3:**
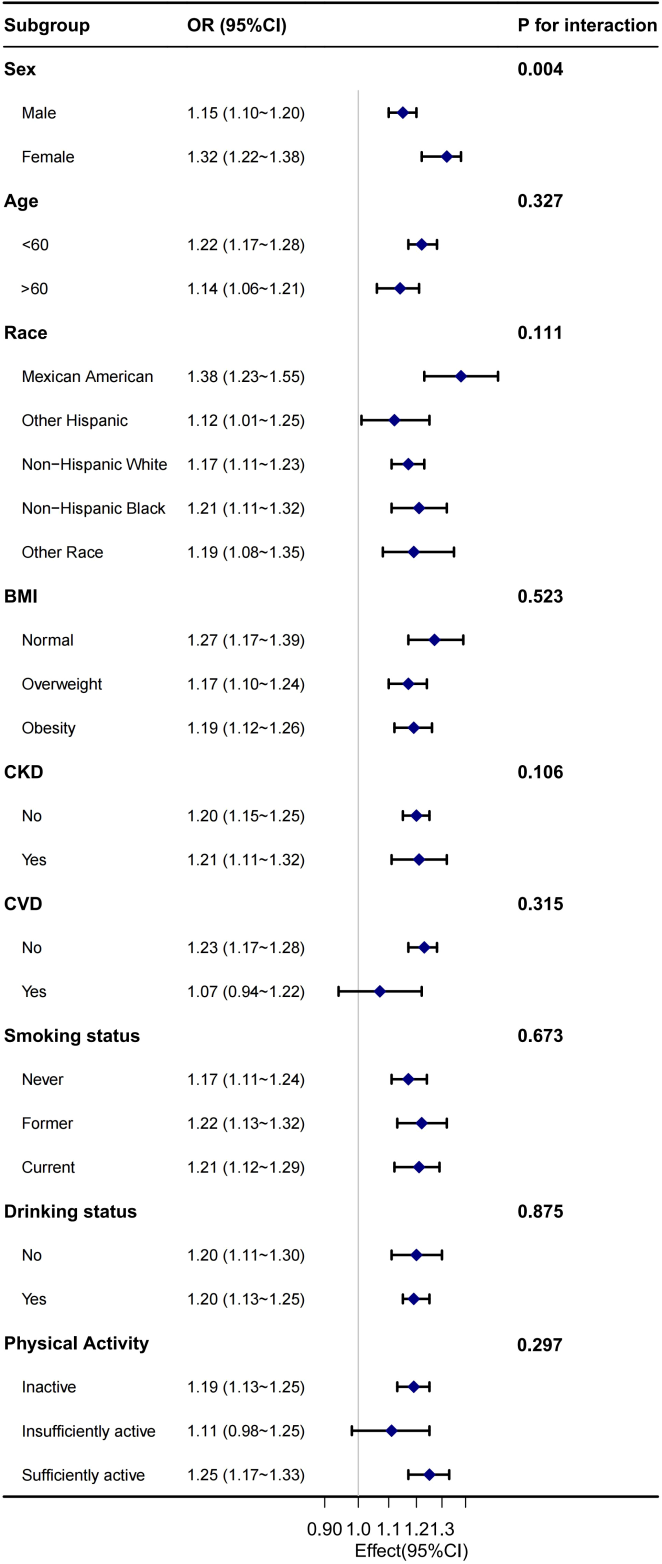
Stratified analysis for the association between NHHR and the risk of diabetes/prediabetes 539 in the male hypertension population.

Stratified analysis revealed that the correlation between NHHR and diabetes/prediabetes remained consistent across most subgroups, including age, race, BMI, CKD, CVD, smoking status, alcohol consumption, and PA. However, sex significantly modified the correlation between NHHR and diabetes/prediabetes in the hypertensive population, indicating a potential sex difference. Notably, the positive association between NHHR and the occurrence of diabetes and prediabetes was stronger in the female population compared to in male population (**Figure 3**). In the sensitivity analysis, after excluding individuals with missing baseline covariates, 7,103 individuals remained. Following adjustment for potential confounding factors, the relationship between NHHR and both diabetes and prediabetes remained consistent with the main analysis (**Table 7**).

**Table 7:** Multivariate Logistic analysis of association between NHHR and the risk of diabetes/prediabetes after excluding participants with any missing covariate values at baseline.

Variable	Model 0		Model 1		Model 2	
	OR (95%CI)	*P*-value	OR (95%CI)	*P*-value	OR (95%CI)	*P*-value
NHHR continues	1.15 (1.11~1.19)	<0.001	1.31 (1.25~1.36)	<0.001	1.20 (1.15~1.25)	<0.001
NHHR quartiles						
Q1	Reference		Reference		Reference	
Q2	1.23 (1.08~1.41)	0.002	1.39 (1.21~1.61)	<0.001	1.16 (0.99~1.35)	0.063
Q3	1.40 (1.23~1.60)	<0.001	1.69 (1.48~1.92)	<0.001	1.39 (1.29~1.63)	<0.001
Q4	1.74 (1.52~2.01)	<0.001	2.46 (2.16~2.62)	<0.001	1.88 (1.68~2.04)	<0.001
*p* for trend	<0.001		<0.001		<0.001	

OR, odds ratio; 95% CI, 95% confidence interval.

Model 1: Non-adjusted.

Model 2: Sex, age, education, race, marital status, and PIR.

Model 3: Model 2 + BMI, Waist circumference, smoking status, drinking habits, physical activity, Uric acid, CVD, CKD.

### 3.6 Predictive ability

**Figure 4:**
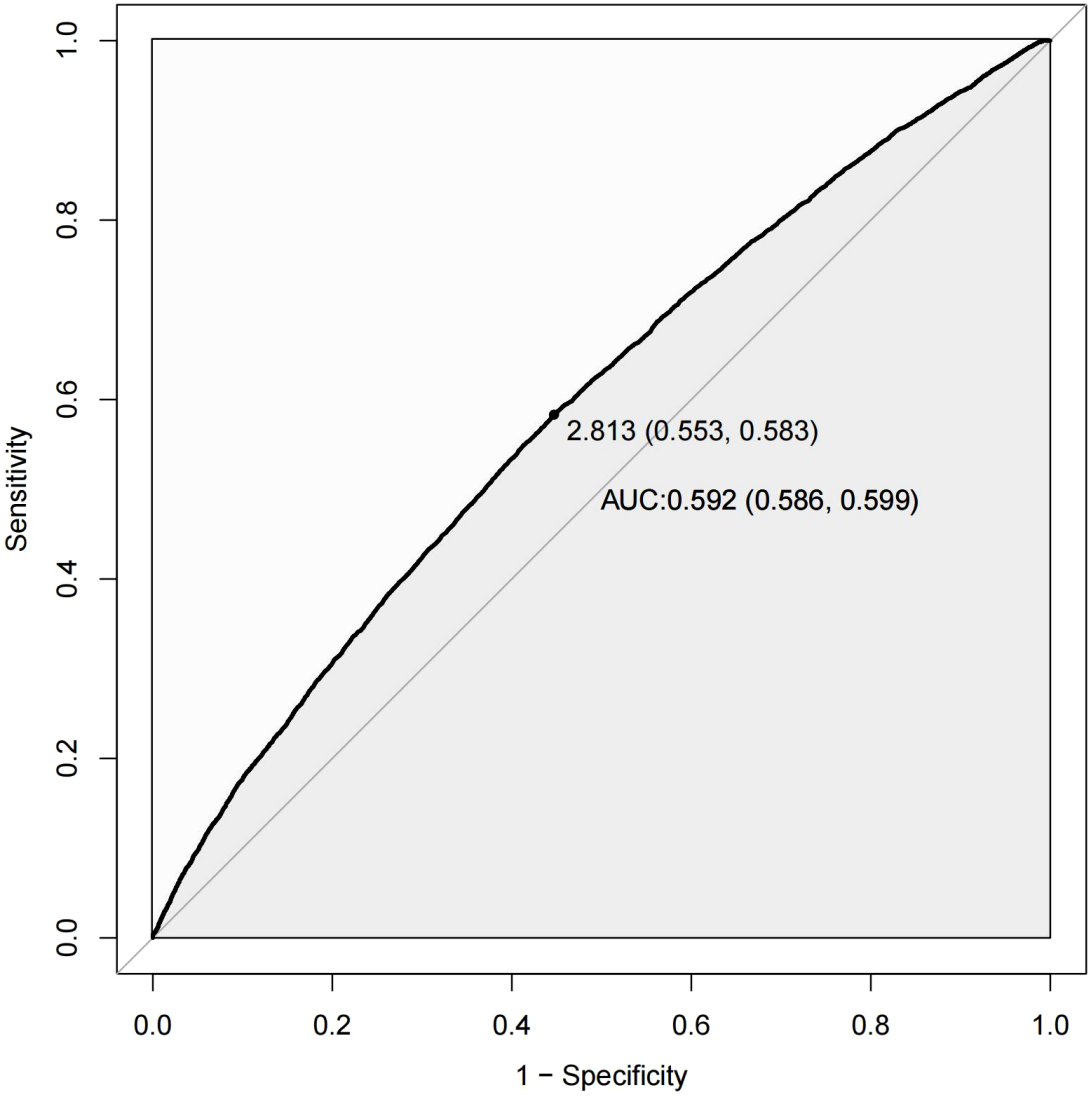
ROC curves of NHHR for predicting diabetes and prediabetes.

**Figure 4** presents the ROC curve of the NHHR for predicting diabetes and prediabetes. The area under the curve (AUC) for NHHR was 0.592, with corresponding sensitivity and specificity values of 0.583 and 0.553 for diabetes and prediabetes, respectively. The area under the curve (AUC) for NHHR was 0.592, with corresponding sensitivity and specificity values of 0.583 and 0.553 for diabetes and prediabetes, respectively (**Table 8**).

**Table 8:** Comparison of NHHR in predicting diabetes and prediabetes.

Variable	AUC (95%CI)	Best threshold	Sensitivity	Specificity
NHHR	0.592 (0.586, 0.599)	2.813	0.583	0.553

AUC, area under the curve; NHHR, non-high-density lipoprotein cholesterol to high-density lipoprotein cholesterol ratio.

## 4 Discussion

We conducted a nationally representative large-scale cross-sectional study using the NHANES dataset to investigate the association between NHHR and the risk of diabetes/prediabetes in individuals with hypertension in the U.S. This study identified a strong and stable positive correlation between NHHR and the risk of both the conditions. After adjusting for confounding factors, NHHR was found to be an independent risk factor for the progression of prediabetes and diabetes in patients with hypertension. For each unit increase in the NHHR, the likelihood of developing diabetes or prediabetes increased by 21%.

Diabetes mellitus, a complex metabolic disorder, is primarily characterized by dysregulated lipid metabolism and obesity. The newly developed comprehensive index, NHHR, has emerged as a cost-effective and easily accessible indicator for assessing atherosclerotic lipid composition (23). It not only predicts the risk of atherosclerosis-related diseases more effectively but also provides indirect insight into the homeostasis between pro-atherogenic and anti-atherogenic lipoproteins (24, 25). Dyslipidemia during atherosclerosis independently affects the occurrence of diabetes. Patients with diabetes have at least double the cardiovascular risk, and this increased risk is the result of multiple risk factors, including dyslipidemia (26). The likelihood of developing type 2 diabetes is significantly related to high non-HDL-C or low HDL-C (27–29). A study conducted in South Korea demonstrated that low HDL cholesterol levels are consistently associated with an increased risk of type 2 diabetes (30). Additionally, research highlighted that non-HDL cholesterol reflects the entire composition of atherogenic lipoproteins rather than LDL cholesterol, positioning non-HDL cholesterol as a crucial predictive indicator of cardiovascular risk in patients with dyslipidemia associated with diabetes (31). The NHHR encompasses both the protective effects of HDL-C and the risk factors related to non-HDL-C, thus providing a more comprehensive view of individual lipid metabolism and more accurately reflecting complex lipid metabolism in patients with diabetes. Research on the correlation between the NHHR and diabetes as well as prediabetes remains limited. A longitudinal cohort study conducted at Murakami Memorial Hospital in Japan indicated a positive independent relationship between the NHHR and diabetes risk (23). When the NHHR reached approximately 2.74, the hazard ratio (HR) for diabetes risk was approximately 1. This aligns with our findings, which revealed a nonlinear link between NHHR and the risk of developing diabetes in patients with hypertension and prediabetes. Our curve-fitting analysis suggested that when the NHHR exceeded 2.807, the risk of diabetes gradually increased. Additionally, Yang et al. found that in individuals over 45 years of age with a BMI greater than 24.0, higher NHHR levels significantly elevated the risk of developing diabetes (32). The large population of prediabetic individuals, who are at a high risk of progressing to diabetes, underscores the importance of understanding the NHHR in this context. Our study established a positive link between the NHHR and both diabetes and prediabetes. However, the connection was stronger in women than in men. Further analysis revealed that the relationship between the NHHR and outcomes for men followed a nonlinear pattern, whereas for women, it was linear.

However, the exact mechanisms underlying this association remain unclear. Several potential mechanisms include the following. First, HDL-C possesses various potential anti-diabetic properties, such as enhancing insulin secretion, promoting β-cell protection, and alleviating insulin resistance (33). Apolipoprotein A-I (apoA-I), the major apolipoprotein component of HDL-C (34), facilitates the uptake of cholesterol and phospholipids through interactions with ATP-binding cassette transporter A1 (ABCA1). Drew et al. found that infusing recombinant HDL-C prepared with apoA-I and soybean phosphatidylcholine in patients with type 2 diabetes enhanced insulin secretion from pancreatic β-cells and increased glucose uptake in skeletal muscle, resulting in lower blood glucose levels (35). *In vitro* studies have shown that HDL can protect and preserve β-cell function (36). Both lipid-free and lipid-associated forms of apoA-I and apoA-II increase insulin synthesis and glucose-stimulated insulin secretion by upregulating the expression of Pdx1, thereby preserving β-cell function and reducing the harmful effects of activated T-cells in diabetes (37). Additionally, an elevated NHHR indicates peripheral cholesterol deposition and accumulation, which play crucial roles in determining the physicochemical properties and functions of cell membranes. Cholesterol accumulation alters the composition of lipid rafts and membrane fluidity, which reduces glucose transporter membrane levels, increases the retention of glucokinase in insulin granules, and alters the spatial organization of L-type voltage-gated calcium channels, ultimately resulting in diminished insulin secretion (38). Type 2 diabetes is characterized by lipid abnormalities, including the accumulation of cholesterol and fatty acids in pancreatic β-cells, exacerbated by overexpression of sterol regulatory element-binding protein 2 (SREBP-2), which leads to severe cholesterol accumulation and severe impairment of cellular function (39). Impaired function of membrane transport proteins such as ABCA1, which is involved in clearing excess cholesterol, results in β-cell dysfunction and reduced insulin secretion (40). Dysregulation of lipoprotein metabolism in type 2 diabetes is ultimately linked to plaque formation and the accumulation of oxidized LDL during atherosclerosis. Elevated non-HDL-C is often linked to a chronic low-grade inflammatory state, in which non-HDL-C particles (such as LDL and VLDL) and free fatty acids directly activate immune cells, particularly macrophages and T cells (41). These immune cells release proinflammatory cytokines and chemokines, further promoting local and systemic inflammation. Insulin resistance and metabolic disturbances in diabetes contribute to oxidative stress, activate pro-inflammatory transcription factors like NF-κB, and mediate cytokine release. Oxidative stress generates a cytotoxic inflammatory environment in islets, specifically damaging β-cells. The combination of chronic oxidative stress and inflammation in the islet microenvironment contributes to the destruction of β-cells by M1-like macrophages and autoreactive T-cell responses. When the non-HDL-C to HDL-C ratio increases, the body’s protective mechanisms are impaired, causing an imbalance in lipid metabolism. Additionally, a decrease in the level of HDL-C tends to be accompanied by higher levels of non-HDL-C, which exacerbates the risk of diabetes (42, 43).

Our sex-stratified sensitivity analyses and subgroup analyses suggested a sex difference in the association between the NHHR and the risk of diabetes and prediabetes, with women being more likely to develop these conditions than men. This phenomenon may be related to the depletion of estrogen levels in adult women. Estrogen plays a protective role in insulin sensitivity as it can enhance hepatic insulin sensitivity by reducing gluconeogenesis and glycogenolysis. Estrogen also prevents β-cell apoptosis, reduces pro-inflammatory signaling, and improves insulin activity. Thus, the higher levels of visceral fat combined with lower endogenous estrogen in men may be associated with increased insulin resistance compared to premenopausal women, potentially contributing to the observed sex differences in cardiovascular disease. A reduction in estrogen levels after menopause may result in disturbances in glucose and lipid metabolism, thereby increasing the risk of diabetes (44). Furthermore, women tend to accumulate subcutaneous fat in areas such as the abdomen, hips, and thighs, whereas men typically store visceral fat in the abdominal region (45). Postmenopausal women are more likely to experience an increase in visceral fat, which promotes insulin resistance and inflammatory responses, further elevating their risk of developing diabetes.

### 4.1 Advantages and limitations of the study

This study has several strengths. It utilized national-level data and offered a large sample size and detailed participant information, strengthening the representativeness and stability of the findings. Additionally, we adjusted for confounding factors to provide the best evidence for an association between NHHR and diabetes/prediabetes. Weighting adjustments were applied according to the NHANES analytical guidelines, and sensitivity analyses were performed with results consistent with the primary analysis, thereby increasing the credibility of the study. However, this study has some limitations. First, the NHANES database includes only baseline information at the time of TC and HDL-C measurement, which may not capture dynamic changes over time. Second, this was a retrospective study; therefore, a causal relationship between the NHHR and diabetes/prediabetes could not be established. Although we adjusted for many known confounders, the influence of unmeasured variables, such as dietary habits and stress levels, cannot be excluded. Therefore, further randomized controlled trials and longitudinal studies are warranted to validate our findings.

### 4.2 Conclusions

Our study established a significant and independent correlation between NHHR and diabetes/prediabetes risk in adults with hypertension. Specifically, a non-linear positive relationship was observed. Notably, this association was stronger in women than in men. Our study provides a basis and valuable reference for further investigations into the associations between NHHR and diabetes/prediabetes risk. Thus, NHHR may play a significant role in the early detection and management of populations at risk for both the conditions.

## Data Availability

The original contributions presented in the study are included in the article/**Supplementary Material**. Further inquiries can be directed to the corresponding author.

## References

[1] Saeedi P Petersohn I Salpea P Malanda B Karuranga S Unwin N. Global and regional diabetes prevalence estimates for 2019 and projections for 2030 and 2045: Results from the International Diabetes Federation Diabetes Atlas, 9 edition. Diabetes Res Clin Pract. (2019) 157:107843. doi: 10.1016/j.diabres.2019.107843 31518657

[2] Lin X Xu Y Pan X Xu J Ding Y Sun X. Global, regional, and national burden and trend of diabetes in 195 countries and territories: an analysis from 1990 to 2025. Sci Rep. (2020) 10:14790. doi: 10.1038/s41598-020-71908-9 32901098 PMC7478957

[3] Edwards CM Cusi K. Prediabetes: A worldwide epidemic. Endocrinol Metab Clin North Am. (2016) 45:751–64. doi: 10.1016/j.ecl.2016.06.007 27823603

[4] Tabák AG Herder C Rathmann W Brunner EJ Kivimäki M. Prediabetes: a high-risk state for diabetes development. Lancet. (2012) 379:2279–90. doi: 10.1016/S0140-6736(12)60283-9 PMC3891203 22683128

[5] Johnson RJ Nakagawa T Sanchez-Lozada LG Shafiu M Sundaram S Le M. Sugar, uric acid, and the etiology of diabetes and obesity. Diabetes. (2013) 62:3307–15. doi: 10.2337/db12-1814 PMC3781481 24065788

[6] Xu Z Liu D Zhai Y Tang Y Jiang L Li L. Association between the oxidative balance score and all-cause and cardiovascular mortality in patients with diabetes and prediabetes. Redox Biol. (2024) 76:103327. doi: 10.1016/j.redox.2024.103327 39186882 PMC11389538

[7] Sánchez E Betriu À López-Cano C Hernández M Fernández E Purroy F. ILERVAS project collaborators. Characteristics of atheromatosis in the prediabetes stage: a cross-sectional investigation of the ILERVAS project. Cardiovasc Diabetol. (2019) 18:154. doi: 10.1186/s12933-019-0962-6 31729979 PMC6857207

[8] Qi X Wang S Huang Q Chen X Qiu L Ouyang K. The association between non-high-density lipoprotein cholesterol to high-density lipoprotein cholesterol ratio (NHHR) and risk of depression among US adults: A cross-sectional NHANES study. J Affect Disord. (2024) 344:451–7. doi: 10.1016/j.jad.2023.10.064 37838268

[9] Kim SW Jee JH Kim HJ Jin SM Suh S Bae JC. Non-HDL-cholesterol/HDL-cholesterol is a better predictor of metabolic syndrome and insulin resistance than apolipoprotein B/apolipoprotein A1. Int J Cardiol. (2013) 168:2678–83. doi: 10.1016/j.ijcard.2013.03.027 23545148

[10] Zhang N Hu X Zhang Q Bai P Cai M Zeng TS. Non-high-density lipoprotein cholesterol: High-density lipoprotein cholesterol ratio is an independent risk factor for diabetes mellitus: Results from a population-based cohort study. J Diabetes. (2018) 10:708–14. doi: 10.1111/1753-0407.12650 29437292

[11] Xiong B Li Z Zhang S Wang Z Xie Y Zhang M. Association between non-high-density lipoprotein cholesterol to high-density lipoprotein cholesterol ratio (NHHR) and the risk of post-stroke depression: A cross-sectional study. J Stroke Cerebrovasc Dis. (2024) 33:107991. doi: 10.1016/j.jstrokecerebrovasdis.2024.107991 39227001

[12] Chen T Cheng Y Song Z Zhang G Zeng T Chao H. Association between non-high-density lipoprotein cholesterol to high-density lipoprotein cholesterol ratio (NHHR) and kidney stone: Evidence from NHANES 2007-2018. BMC Public Health. (2024) 24:1818. doi: 10.1186/s12889-024-19265-4 38978025 PMC11232242

[13] Luo X Ye J Xiao T Yi T. Exploration of the association of a lipid-related biomarker, the non-high-density lipoprotein cholesterol to high-density lipoprotein cholesterol ratio (NHHR), and the risk of breast cancer in American women aged 20 years and older. Int J Surg. (2024) 110:5939–41. doi: 10.1097/JS9.0000000000001700 PMC11392120 38781036

[14] Carr SS Hooper AJ Sullivan DR Burnett JR. Non-HDL-cholesterol and apolipoprotein B compared with LDL-cholesterol in atherosclerotic cardiovascular disease risk assessment. Pathology. (2019) 51:148–54. doi: 10.1016/j.pathol.2018.11.006 30595507

[15] Taskinen MR. Diabetic dyslipidaemia: From basic research to clinical practice. Diabetologia. (2003) 46:733–49. doi: 10.1007/s00125-003-1111-y 12774165

[16] Zhang X Xing L Jia X Pang X Xiang Q Zhao X. Comparative lipid-lowering/increasing efficacy of 7 statins in patients with dyslipidemia, cardiovascular diseases, or diabetes mellitus: Systematic review and network meta-analyses of 50 randomized controlled trials. Cardiovasc Ther. (2020) 2020:3987065. doi: 10.1155/2020/3987065 32411300 PMC7201823

[17] Lin D Qi Y Huang C Wu M Wang C Li F. Associations of lipid parameters with insulin resistance and diabetes: A population-based study. Clin Nutr. (2018) 37:1423–9. doi: 10.1016/j.clnu.2017.06.018 28673690

[18] Ley SH Harris SB Connelly PW Mamakeesick M Gittelsohn J Wolever TM. Utility of non-high-density lipoprotein cholesterol in assessing incident type 2 diabetes risk. Diabetes Obes Metab. (2012) 14:821–5. doi: 10.1111/j.1463-1326.2012.01607.x 22510237

[19] Ahluwalia N Dwyer J Terry A Moshfegh A Johnson C. Update on NHANES dietary data: Focus on collection, release, analytical considerations, and uses to inform public policy. Adv Nutr. (2016) 7:121–34. doi: 10.3945/an.115.009258 PMC4717880 26773020

[20] Carey RM Whelton PK 2017 ACC/AHA hypertension guideline writing committee. Prevention, detection, evaluation, and management of high blood pressure in adults: Synopsis of the 2017 american college of cardiology/american heart association hypertension guideline. Ann Intern Med. (2018) 168:351–8. doi: 10.7326/M17-3203z 29357392

[21] Zhao S Yu S Chi C Fan X Tang J Ji H. Association between macro- and microvascular damage and the triglyceride glucose index in community-dwelling elderly individuals: the Northern Shanghai Study. Cardiovasc Diabetol. (2019) 18:95. doi: 10.1186/s12933-019-0898-x 31345238 PMC6657056

[22] Cederholm T Bosaeus I Barazzoni R Bauer J Van Gossum A Klek S. Diagnostic criteria for malnutrition - an ESPEN consensus statement. Clin Nutr. (2015) 34:335–40. doi: 10.1016/j.clnu.2015.03.001 25799486

[23] Sheng G Liu D Kuang M Zhong Y Zhang S Zou Y. Utility of non-high-density lipoprotein cholesterol to high-density lipoprotein cholesterol ratio in evaluating incident diabetes risk. Diabetes Metab Syndr Obes. (2022) 15:1677–86. doi: 10.2147/DMSO.S355980 PMC9166911 35669362

[24] Han M Li Q Qie R Guo C Zhou Q Tian G. Association of non-HDL-C/HDL-C ratio and its dynamic changes with incident type 2 diabetes mellitus: The Rural Chinese Cohort Study. J Diabetes Complications. (2020) 34:107712. doi: 10.1016/j.jdiacomp.2020.107712 32919864

[25] Hsu SH Jang MH Torng PL Su TC. Positive association between small dense low-density lipoprotein cholesterol concentration and biomarkers of inflammation, thrombosis, and prediabetes in non-diabetic adults. J Atheroscler Thromb. (2019) 26:624–35. doi: 10.5551/jat.43968 PMC6629751 30587667

[26] Márk L Dani G. Diabeteses dyslipidaemia és atherosclerosis [Diabetic dyslipidaemia and the atherosclerosis. Orv Hetil. (2016) 157:746–52. doi: 10.1556/650.2016.30441 27133274

[27] Sokooti S Flores-Guerrero JL Kieneker LM Heerspink HJL Connelly MA Bakker SJL. HDL particle subspecies and their association with incident type 2 diabetes: the PREVEND study. J Clin Endocrinol Metab. (2021) 106:1761–72. doi: 10.1210/clinem/dgab075 PMC8118359 33567068

[28] Sokooti S Flores-Guerrero JL Heerspink HJL Connelly MA Bakker SJL Dullaart RPF. Triglyceride-rich lipoprotein and LDL particle subfractions and their association with incident type 2 diabetes: the PREVEND study. Cardiovasc Diabetol. (2021) 20:156. doi: 10.1186/s12933-021-01348-w 34321006 PMC8320057

[29] Krentz AJ. Lipoprotein abnormalities and their consequences for patients with type 2 diabetes. Diabetes Obes Metab. (2003) 5:S19–27. doi: 10.1046/j.1462-8902.2003.0310.x 14984018

[30] Lee MK Han K Heo J Byungpyo K. 1279-P: Cumulative exposure to high triglyceride–to–HDL cholesterol ratio and risk of type 2 diabetes in young adults. Diabetes. (2024) 73:5. doi: 10.2337/db24-1279-P 38118001

[31] Anne L. Peters; clinical relevance of non-HDL cholesterol in patients with diabetes. Clin Diabetes 1 January. (2008) 26:3–7. doi: 10.2337/diaclin.26.1.3

[32] Yu Y Wu Y Su X. Associations between non -HDL -c/HDL-c, its dynamics changes and incident type 2 diabetes mellitus in a prospective cohort study. Modern Preventive Meacine. (2022) 49(7):1175–80. doi: 10.3969/j.issn.1003-8507.2022.7.xdyfyx202207005

[33] Lui DTW Tan KCB. High-density lipoprotein in diabetes: Structural and functional relevance. J Diabetes Investig. (2024) 15:805–16. doi: 10.1111/jdi.14172 PMC11215696 38416054

[34] Kontush A Lindahl M Lhomme M Calabresi L Chapman MJ Davidson WS. Structure of HDL: Particle subclasses and molecular components. Handb Exp Pharmacol. (2015) 224:3–51. doi: 10.1007/978-3-319-09665-0_1 25522985

[35] Drew BG Duffy SJ Formosa MF Natoli AK Henstridge DC Penfold SA. High-density lipoprotein modulates glucose metabolism in patients with type 2 diabetes mellitus. Circulation. (2009) 119:2103–11. doi: 10.1161/CIRCULATIONAHA.108.843219 19349317

[36] Nilsson O Del Giudice R Nagao M Grönberg C Eliasson L Lagerstedt JO. Apolipoprotein A-I primes beta cells to increase glucose stimulated insulin secretion. Biochim Biophys Acta Mol basis Dis. (2020) 1866:165613. doi: 10.1016/j.bbadis.2019.165613 31765698

[37] Wang X Sterr M Ansarullah Burtscher I Böttcher A Beckenbauer J. Point mutations in the PDX1 transactivation domain impair human β-cell development and function. Mol Metab. (2019) 24:80–97. doi: 10.1016/j.molmet.2019.03.006 30930126 PMC6531841

[38] Gondré-Lewis MC Petrache HI Wassif CA Harries D Parsegian A Porter FD. Abnormal sterols in cholesterol-deficiency diseases cause secretory granule malformation and decreased membrane curvature. J Cell Sci. (2006) 119:1876–85. doi: 10.1242/jcs.02906 16636072

[39] Xia F Xie L Mihic A Chen Y Gaisano HY Tsushima RG. Inhibition of cholesterol biosynthesis impairs insulin secretion and voltage-gated calcium channel function in pancreatic beta-cells. Endocrinology. (2008) 149:5136–45. doi: 10.1210/en.2008-0161 18599549

[40] Kruit JK Kremer PHC Dai L Tang R Ruddle P de Haan W. Cholesterol efflux via ATP-binding cassette transporter A1 (ABCA1) and cholesterol uptake via the LDL receptor influences cholesterol-induced impairment of beta cell function in mice. Diabetologia. (2010) 53:1110–9. doi: 10.1007/s00125-010-1691-2 20229095

[41] Chen X Xie N Feng L Huang Y Wu Y Zhu H. Oxidative stress in diabetes mellitus and its complications: From pathophysiology to therapeutic strategies. Chin Med J (Engl). (2025) 138:15–27. doi: 10.1097/CM9.0000000000003230 39503316 PMC11717531

[42] Rütti S Ehses JA Sibler RA Prazak R Rohrer L Georgopoulos S. Low- and high-density lipoproteins modulate function, apoptosis, and proliferation of primary human and murine pancreatic beta-cells. Endocrinology. (2009) 150:4521–30. doi: 10.1210/en.2009-0252 19628574

[43] Abderrahmani A Niederhauser G Favre D Abdelli S Ferdaoussi M Yang JY. Human high-density lipoprotein particles prevent activation of the JNK pathway induced by human oxidised low-density lipoprotein particles in pancreatic beta cells. Diabetologia. (2007) 50:1304–14. doi: 10.1007/s00125-007-0642-z 17437081

[44] Meyer MR Clegg DJ Prossnitz ER Barton M. Obesity, insulin resistance and diabetes: sex differences and role of oestrogen receptors. Acta Physiol (Oxf). (2011) 203:259–69. doi: 10.1111/j.1748-1716.2010.02237.x PMC3110567 21281456

[45] Pouliot MC Després JP Lemieux S Moorjani S Bouchard C Tremblay A. Waist circumference and abdominal sagittal diameter: Best simple anthropometric indexes of abdominal visceral adipose tissue accumulation and related cardiovascular risk in men and women. Am J Cardiol. (1994) 73:460–8. doi: 10.1016/0002-9149(94)90676-9 8141087

